# In COVID We Trust: The Impact of The Pandemic on Religiousness—Evidence from Italian Regions

**DOI:** 10.1007/s10943-023-01755-1

**Published:** 2023-02-08

**Authors:** Vincenzo Alfano, Salvatore Ercolano, Gaetano Vecchione

**Affiliations:** 1 DiSEGIM, University of Napoli Parthenope, Naples, Italy; 2 Center for Economic Studies – CESifo, Munich, Germany; 3 Department of Mathematics, Information Sciences and Economics, University of Basilicata, Potenza, Italy; 4 Department of Political Sciences, University of Naples Federico II, Naples, Italy

**Keywords:** COVID-19, Coronavirus, Religious attendance, Mass, Mass streaming

## Abstract

By changing many aspects of everyday life, the COVID-19 pandemic and the social distance policies implemented to face it have affected the behaviour of people all over the world. Has the pandemic also affected people’s approach towards the divine? Previous evidence suggests that prayer searches on the Internet rose during the pandemic and that people tend to rely mainly on intrinsic rather than extrinsic religiousness to cope with adversity. In the present contribution, using a set of panel random effect estimators, we compare the change in religious attendance in Italian regions before and during the pandemic. Our results suggest that there has been an increase in religiousness during the COVID-19 pandemic. Our findings are robust to several specifications of the model and to different estimators. This suggests that people derive more comfort from religious activities during hard times that are characterized by uncertainty.

## Introduction

Beginning in December 2019, a coronavirus infectious disease (COVID-19) rapidly spread across the world. The intensity of the pandemic changed over time and across countries, as did the measures taken to mitigate its effects. Various authorities all over the world put in place different strategies (Piguillem & Shi, 2020), though in Europe at least the response was quite homogeneous (Alfano et al., 2022a).

The policies may be divided into two large groups: measures that are strictly health-related, and those that are non-pharmaceutical. While the first are aimed at strengthening the capacity of the health system to deal with the effects of COVID-19 (and thus include policies such as plans for the expansion of the health workforce, support for companies producing medical supplies, and so on), the latter aim to reduce the probability of individual citizens contracting the virus (and thus this group includes policies such as lockdowns and other social distancing measures).

There is a growing literature in the social sciences aimed at assessing the efficacy of this second kind of policy, usually in terms of the reduction of new COVID-19 cases. Alfano and Ercolano (2020), adopting a cross-country perspective, assess the efficacy of lockdown measures in containing the diffusion of the virus. The authors suggest that, on average, the efficacy of lockdown in reducing new cases lasts from 7 to at least 20 days after implementation. Other scholars, like Sarwal and Sarwal (2020), looking at the Indian case and adopting a within-country perspective, suggest that this efficacy could be different if one looks at different provinces.

Obviously, non-pharmaceutical measures may also have an impact on economic activities. Correia et al. (2020), investigating the implementation of such measures during the fall of 1918, do not find that stricter non-pharmaceutical measures were associated with a larger decline in economic outcomes in the years following the pandemic. More specifically, *ceteris paribus*, cities in which non-pharmaceutical measures were implemented strictly during the pandemic also registered a relative increase in economic activity from 1919 onwards. In fact, according to the authors, despite the fact that social distancing constrains interactions and thus economic activities, during a pandemic households tend to reduce their consumption and labour supply in order to reduce the probability of being infected. These authors’ results support the idea that such measures are able to reduce disease transmission without exacerbating the negative economic impact.

Following this framework, Kaplan et al. (2020) try to quantify the trade-off between saving lives and worsening economic outcomes due to the implementation of a lockdown, and the related distributional effects due to COVID-19. According to the authors, the pandemic may have different impacts on different individuals, due to different degrees of economic exposure triggered by the pandemic. Exposure could depend on how their jobs are linked with the production of social goods, as well as the access to government transfers (the fact that the effects of the two are intertwined, as suggested by Alfano et al. (2022b), seems to be especially important). Their findings suggest that the most exposed individuals are also those that are most vulnerable. Moreover, in the absence of lockdown measures, the second and following waves of the pandemic could be associated with a more important economic cost, especially with regard to the most vulnerable individuals.

In any case, aside from focusing on the impact of policies, it is important to study the social causes that may contribute to spreading the contagion and its effects on the population. Another branch of this literature is emerging, and is devoted to investigating the effects of such exogenous shocks, with several different outcomes.

It is easy to imagine that the fear of a pandemic, as well as the imposition of social distancing and limitations of movement due to lockdown measures, other than being affected by individual characteristics, such as social capital (Alfano & Ercolano, 2021; Alfano, 2022a) or work ethics (Alfano, 2022b), may also have generated a swift and significant modification in individuals’ behaviour.

More precisely, several scholars have tried to analyse these changes, and some of them have used statistics from web searches. Among them, Brodeur et al. (2020), using Google Trends data for Europe and the US, found an increase in search intensity for terms such as boredom, loneliness, worry, and sadness: this suggests a possible negative effect of lockdown on people’s mental health.

Nevertheless, according to other scholars, lockdown measures may also have some positive impact. For example, Ding et al. (2020), using Google Trends data, found an increase in population-level interest in engagement with physical activity. According to the authors, this may be due to a large availability of discretionary time, as well as the recommendations coming from media, governments, and health authorities.

The combination of the fear of the pandemic and the requirement to stay at home could also push people to turn to religion as a means to overcome a situation characterized by material and immaterial adversity. In their seminal contribution, Norris and Inglehart (2004) propose and discuss their theory of existential insecurity, explaining why countries with lower rates of human development are generally more religious. Religion is an important resource for dealing with existential insecurity, and religious communities have already been suggested to play a role in this relationship (Lee et al., 2022); therefore, we should expect that amid the COVID-19 crisis, facing increased uncertainty and existential insecurity, people turn more towards religion.

In this interesting and potentially fruitful framework, Bentzen (2020) finds that during March 2020, Google searches for prayer rose to the highest level ever recorded. This result seems to be quite common all over the world, despite some variations that seem to occur among different religions, and suggests that ‘when faced with uncertainty and adversity, humans tend to use religion for comfort and explanation’ (*ibid.*, p. 73).

Indeed, a common finding in the literature is that people use their religion to cope with adversity (Cohen & Wills, 1985; Norenzayan & Hansen, 2006; Pargament, 2001; Park et al., 1990; Williams et al., 1991). This literature, to which the current study aims to contribute, links religiousness—which is a cultural value with very significant implications for economic outcomes—to the need to cope with a worldwide calamity. As Bentzen (2020) highlights: ‘If the COVID-19 pandemic strengthens religion permanently, this may have socio-economic consequences later on’.

Nevertheless, it seems to us that the increased interest in prayers on the Internet is insufficient as a basis for any conclusions about a general rise of interest in religion. Indeed, this is possible only related to the larger availability of discretionary time during the pandemic, a consequence highlighted by Ding et al. (2020). On the other hand, it could also be a result that is inflated by a search for alternative sources of spiritual comfort driven by the impossibility of participating in person in religious ceremonies, due to social distancing policies.

Furthermore, accepting the increase in prayer found by Bentzen (2020), as we have already explained it is of interest to the social sciences to understand whether the pandemic causes a rise in extrinsic religiousness as well as intrinsic religiousness. All these points lead to another research question: has the coronavirus crisis also caused an increase in the attendance of organized religious rites? The cost-opportunity of investing in personal prayer is much lower compared to that of investing in attending an organized religious rite.

Did areas more affected by COVID-19 see increased levels of religious attendance among their populations? It has been suggested that people tend to rely mainly on intrinsic religiousness (such as private prayer) rather than extrinsic religiousness (for instance, churchgoing) in order to cope with adversity (Bentzen, 2019; Johnson & Spilka, 1991; Pargament, 2001). Does this also apply to the COVID-19 case?

Accordingly, the present contribution aims to investigate the relationship between the outbreak of the COVID-19 pandemic and the rise in the search for comfort from attending religious rites. In order to address this research topic, we rely on a quantitative approach, investigating the individual behaviour observed in Italian regions during the COVID-19 pandemic, in the 10 weeks between mid-March and mid-May, during which a ban was in place on attending Mass in person.

The Italian case could be the ideal setting to test hypotheses on the impact of COVID-19 on religious aptitudes, for two principal reasons. First of all, Italy is well known for the wide diffusion among its population of Catholicism (Knill & Preidel, 2015); this makes the country highly suitable for investigation of the religion-related issue without the possibility of biases due to different coexisting religious mandates, aptitudes, and habits, which may easily muddy the results. It is also worth noting that previous research has found that the surge in average religiousness after a natural disaster, common to all major religions (Bentzen, 2020), is lower than average among Catholics (Bentzen, 2019).

This suggests that a finding taken from a country with a mostly Catholic population is likely extendable to other religions and that a bigger magnitude in a religiously more heterogeneous population is likely to be measured. Furthermore, Italy is among the countries that have suffered the most from COVID-19 due to the high levels of contagion that were registered at the beginning of 2020, and it is thus a good setting in which to test COVID-related issues, also because the levels of contagion registered are very heterogeneous among the different Italian regions.

Indeed, after the first outbreak registered in Wuhan, the second country to be severely hit by the pandemic in the spring of 2020 was Italy, which was a very particular case in Europe at the time. Despite the relatively small geographical extension of the country (at least compared to other countries that experienced a violent burst of the epidemic, such as China and Brazil, for instance), the diffusion of the virus varied widely in different Italian regions.

It is worth noting that when Italy registered the peak of its pandemic around March 21 2020, three northern regions had more than 68% of the total cases. More specifically, on that day Lombardy registered 25,515 total cases (about 47% of the total cases in Italy), Emilia Romagna 6705 cases (12.5%), and Veneto 4617 cases (8.6%); whereas among the southern regions (i.e. those least affected by the pandemic), Calabria in the same day counted just 235 total cases (0.4%), Basilicata 66 cases (0.12%) and Molise 61 cases (0.11%). Despite this heterogeneity, on 11 March 2020 the Italian government decided to adopt a total lockdown across the country to contain the diffusion of the virus.

Following the above, we formulate the following research question:

RQ. Has the COVID-19 pandemic had an impact on people’s extrinsic religiousness?

Have people living in areas that are more affected by a virus outbreak turned more towards religion?

In order to test this hypothesis in a quantitative framework, following a well-established development in applied economics (Choi & Varian, 2009), the present manuscript relies on data extracted from Google Trends. For our aims, we consider it possible to exploit the fact that a lockdown was in place and that there was thus the consequent impossibility, on the one hand, of physically taking part in Mass, and the possibility, on the other, of following Mass via streaming services. This allows us to determine how many people attended Mass in each Italian region during the lockdown period.

Comparing these data with the attendance rates of Italian regions recorded prior to the pandemic, we may derive the impact on religiousness of the spread of COVID-19 infections and deaths related to it. Of course, there are at the same time reasons to believe that the external validity of results found in a largely Catholic country in times of difficulty, such as the Easter of 2020, have a limited generalizability for other periods, countries, or religions. Therefore, the reader should be warned even now about applying our results to other contexts.

The rest of the paper is organized as follows: Sect. 2 describes data and methodology, Sect. 3 discusses the principal results, and in Sect. 4 we provide some conclusions on the basis of the principal findings. Section 5 discusses some limits, while the last section, as usual, concludes.

## Methods

In order to estimate the impact of COVID-19 on religion attendance empirically, we require: a proxy of religiousness for each Italian region, both before the pandemic and during the lockdown; the weekly number of COVID-19 cases and COVID-19-related deaths in each Italian region; and a proxy of accessibility to the Internet in the various Italian regions.

The data about religiousness prior to COVID-19 are gathered from the *Multipurpose Survey on Households: Aspects of Daily Life* published by the Italian Institute of Statistics (ISTAT), and specifically its section on religious practice. It offers data from 2018 (the last year for which regional data were available at the time of writing) about the regional share of people older than 5 that attended a place of worship at least once per week. This is our proxy for the religiousness of the population in the various Italian regions before the COVID-19 pandemic.

To proxy religiousness during the lockdown, meanwhile, we had to apply a different approach. On 12 March Italians faced a complete lockdown, which included several social distancing measures, among them a national ban on celebrating Mass. It is important to note that this was also accepted and supported by the Pope, who ordered all Catholic priests (i.e. the vast majority of religious ministers that celebrate holy rites in Italy) to suspend the celebration of Mass. At the same time, the Pope blessed and sponsored the online streaming of masses, which is something traditionally frowned on by the Catholic Church.

It is notable that the Pope himself celebrated a mass that was streamed online, each morning during the first lockdown period. The Internet is used as a source of information by many people in Italy and all over the world, and it is especially important when it comes to health-related risks (Risk & Dzenowagis, 2001). Google is by far the most popular search engine, and for this reason we believe that the number of searches on Google for the terms *messa streaming* is a good proxy of the interest in following Mass in the different Italian regions during the lockdown.

To test our hypotheses empirically, we built a panel dataset, with weekly data from the Italian regions used as the basic statistical unit of observation. In formal terms, we estimate the following equation:

1 $$\Delta R_{rw} = \alpha + \beta_{1} i_{rw} + \beta_{2} d_{rw} + \beta_{3} DD_{r} + \beta_{4} D{\text{Lom}}_{r} + \beta_{5} DEas + \beta_{6} T + \varepsilon_{rw}$$

where $\Delta R$ is the change in religiousness, proxied through the ratio of attendance of Mass, in the region *r* during week *w*, to religiousness proxied in the same region before COVID-19. As should be clear from Eq. (1), the ratio in religiousness in region *r* is modelled as a function of the total number of COVID-19 cases registered in the week (${i}_{rw}$), and of the deaths related to the virus (${d}_{rw}$), plus a set of control variables.

More precisely, we control for: *DD*, a variable to control for the digital divide, and thus the varying access to high-speed Internet across the Italian regions, which may affect streaming and thus our operationalization of religiousness; *D*Lom, a dichotomous variable signalling whether or not region *r* is Lombardy, which is an outlier among Italian regions and may thus affect the estimates; *Deas*, a dichotomous variable signalling whether or not week *w* is Easter week, given that more people may attend Mass on this important religious celebration; and finally *T* is a set of *N* dichotomous dummies included in the model to control for potential temporary effects in the 10 weeks analysed, and to avoid biases in the estimates due to cyclic or temporary variability (please note that each of these variables assumes the value of 1 for each week included in the analysis, and 0 otherwise, and that the number of weeks included in the analysis is equal to *N* + 1).

**Fig. 1:**
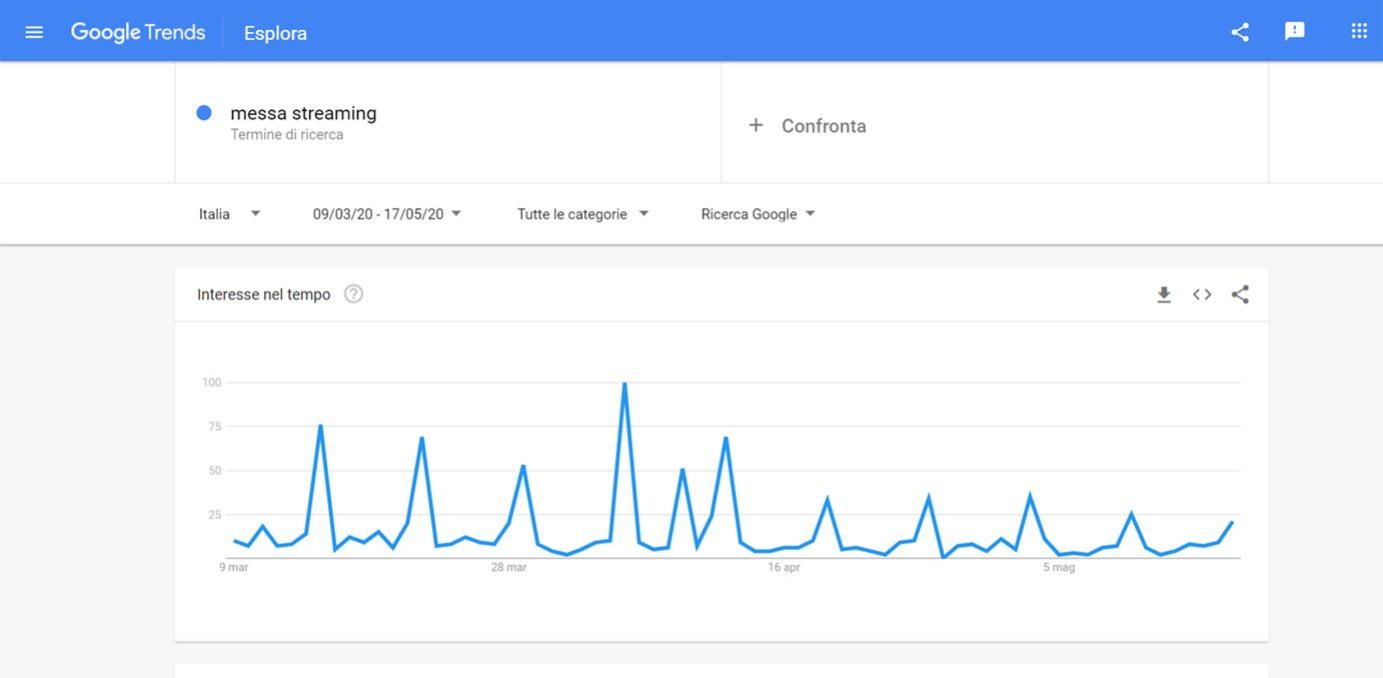
Google Trends for the string messa streaming from March 9 to May 17

Figure 1 seems to provide some preliminary pieces of evidence that suggest the adequacy of our operationalization. It reports the searches for the terms in Italy during the lockdown: the peaks, visible at first glance, correspond to Sundays, the Christian holy day, and that on which Catholics are traditionally required to attend Mass. The greatest peak is on 5 April, Palm Sunday, a day on which the Pope celebrated a special Mass, streamed from Saint Peter’s square, in which he asked God for relief from the pandemic. The subsequent peak registered on the graph, immediately to the right, corresponds with 9 April, Holy Thursday, an important celebration in Catholic tradition in the approach to Easter; and Easter itself was celebrated in 2020 on 12 April, which is the following peak in the graph. In short, the graph seems to confirm that these data should be a good proxy of the demand for religiousness in Italian regions, given their correlation with both Sundays and important Catholic celebrations.

Bearing that in mind, it is important to remark that our analysis operationalizes the demand for religiousness among Italian regions by looking at Google Trends data. Google Trends allows us to inspect the evolution over time and region of Google queries that are related to a specific word or expression. For our analysis, Google Trends results related to the phrase *messa streaming* (Italian for *Mass in streaming*, and very likely to be the terms used for a search online when looking for a Mass in online streaming) were inspected, and the extraction of data was carried out by region. Data extracted through Google Trends are normalized on a 0–100 scale, where 100 represents the region with the greatest frequency of searches on the topic out of the total number of searches in the region, and the other values are relative shares of this 100.

**Fig. 2:**
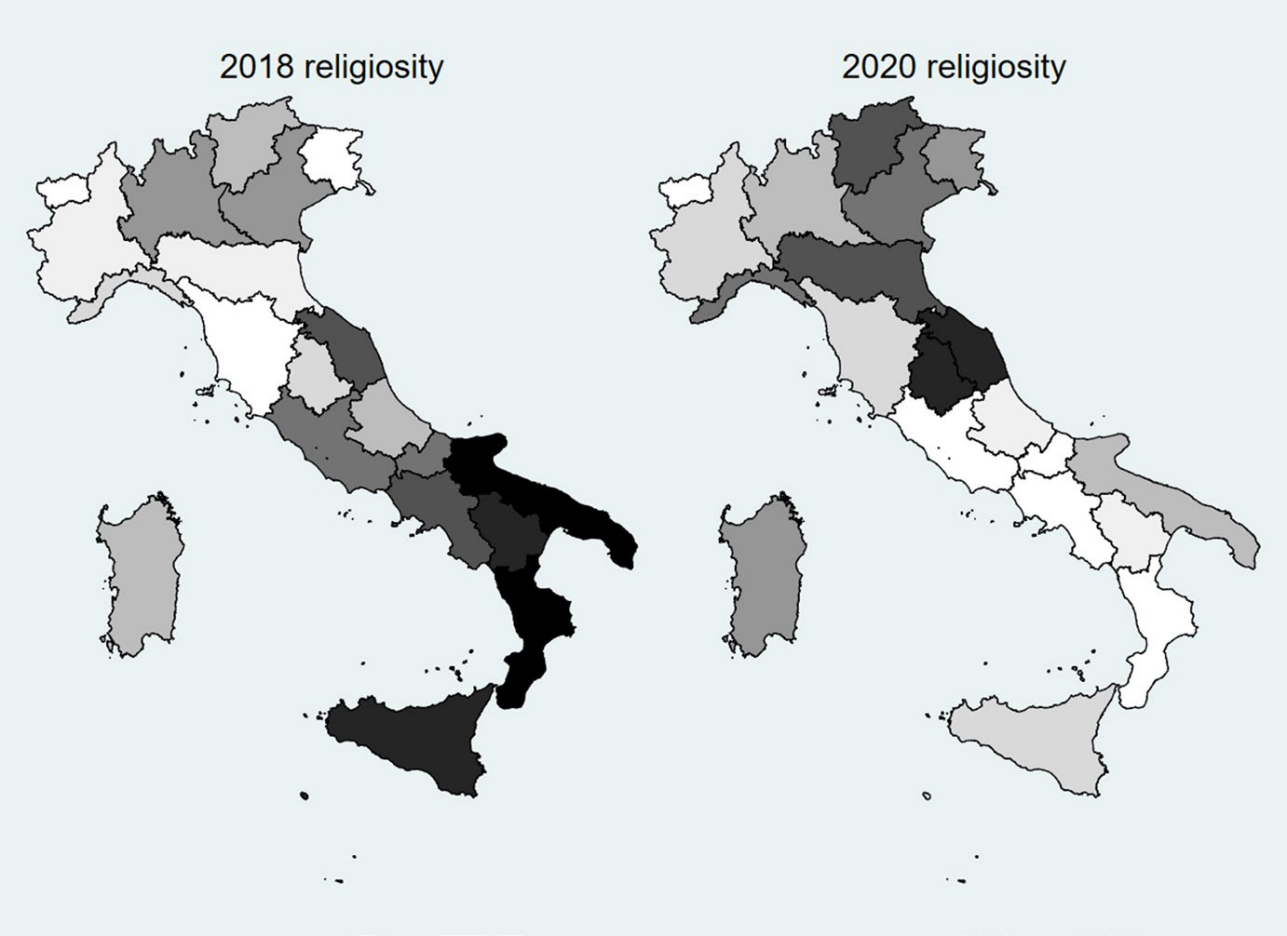
Heat maps comparing religiousness (deciles) in 2018 and in 2020 for different Italian regions

Our proxy of religiousness prior to the pandemic is, as explained, the share of population older than 5 that attended a religious rite at least once per week. We transformed this variable into the same scale used by Google Trends by dividing all the values by the maximum value of the series and multiplying the result by 100. After this normalization, we proceeded to compute our dependent variable, the operationalization of $\Delta {R}_{rw}$: *RatioRel*, computed as the ratio between religiousness in 2020 (numerator) and 2018 (denominator). It is interesting to note, as presented in Fig. 2, that from a preliminary analysis of the regional religiousness it seems to be the case that the pandemic *has* had an important impact on the habits of Italians. Indeed, while in 2018, as anecdotal evidence led us to expect, the vast majority of southern regions belonged to the higher quantile (darker in the figure), in 2020 this was no longer true, and the central and central-northern regions, which were more severely affected by the pandemic, seem to be significantly more religious than they used to be.

Another important set of variables in our empirical analysis is data about COVID-19 infections and deaths. Those are gathered from the Italian Ministry of Health’s dataset, which reports official data for each province and day. Given the importance of attending Mass on Sunday, the Christian holy day, and the so-called weekend effect in the report of COVID-19 cases and deaths (Soukhovolsky et al., 2021), we chose to run our analysis using the week as the basic unit of time. For this reason, we summed the data to obtain total COVID-19 cases and deaths per week per region, from the week ending on Sunday, 15 March, the first week in which Italy faced a total lockdown, to that ending on 17 May, since the ban on celebrating Mass with believers attending in person was lifted from Monday, 18 May. Data are gathered from the latest version of this document while writing (taking us up to 17 May 2020).

This gives a total of 10 weeks observed for 20 different Italian regions. From this source, we computed the variable measuring weekly cases (*Cases*) and weekly deaths (*Deaths*) due to COVID-19 in each Italian region. The literature has questioned the validity of these data (Buonanno et al., 2020; Sabbadini & Morano, 2020), mainly given the presence of asymptomatic cases and the differences in testing policies adopted in the different regions, as well as the quality of the institutions enforcing it. This should not affect our analysis, however, given that we are estimating the impact of the advertised number of cases, which is the number we gathered, on the behaviour of the public, and not the actual number of infected people which, even if higher, may not affect citizens’ behaviour given its lack of publicity. In other words, we are looking for a contemporaneous effect, which should be due to the immediate perception among the public of the number of COVID-19 cases and deaths, because of daily bulletins.

Italian regions are also very heterogeneous in terms of the digital divide, although broadband infrastructure is not a good proxy of this (Quaglione et al., 2020). For this reason, we chose to control for the possibilities of accessing online streaming, which may affect the results given for our operationalization of religiousness during the pandemic, via ISTAT’s *Multipurpose survey on households: aspects of daily life*, and specifically its section on Internet access and availability of technology. More precisely, we employed the share of families with access to a 3G connection per region as a proxy of the digital divide. Furthermore, we decided to control for the observations referred to Lombardy by including in some of the specifications of our model a dummy that allows us to exclude this potential outlier from biasing the results.

Finally, we also considered that Easter week and the week immediately before that may be those in which more Catholics decide to attend Mass via streaming, given the attention devoted in this period of the year to this religious festivity. To control for this effect, which may potentially affect our results, we included a dummy variable equal to 1 for both the fourth and the fifth week, which is that of Easter, and to 0 otherwise.

The final dataset is composed of 10 weekly observations in 20 Italian regions, giving a total of 200 observations. Descriptive statistics of the variables used are provided in Table 1.

**Table 1:** Descriptive statistics

Label	Variable	Obs	Mean	SD	Min	Max
RatioRel	Ratio between religiousness in 2020 and in 2018	200	0.3835341	0.4051973	0	1.957576
Cases	COVID-19 weekly cases in the region	200	48,282.43	93,066.27	55	572,622
Deaths	COVID-19 weekly deaths in the region	200	977.53	2473.629	0	15,519
Digital divide	Share of families in the region with access to 3G connection	200	37.21	3.854111	30.6	48.1
DLom	Dummy equal to 1 if the region is Lombardy, to 0 otherwise	200	0.05	0.2184919	0	1
DEas	Dummy equal to 1 if the week is Easter week or the week before Easter, to 0 otherwise	200	0.2	0.4010038	0	1

Considering that the data have several observations for each *r* and *w*, the natural choice for the estimator to employ is a Feasible–Generalized Least Square (F-GLS) (Aigner & Balestra, 1988; Hsiao, 1986). Our framework requires the use of a random effect estimator, given that we are comparing differences between regions; nevertheless, a Hausman test, presented in Table 2 suggests that also empirically a Random Effects estimator is to be preferred to a Fixed Effects one.

**Table 2:** Hausmant test

Ho: difference in coefficients not systematic
chi2(10) = (*b* −* B*)'[(*V*_*b* −* V*_*B*)^(−1)](*b* −* B*) = 0.16
Prob > chi2 = 1.0000

### Results and Robustness Checks

As can be seen in Table 3, the more cases an Italian region faced during the pandemic, the greater the difference in its 2020 religiousness (related to 2018). This first result, equal in all the specifications of the model, suggests that the COVID-19 pandemic has indeed had an impact on religion. This effect is statistically significant at 10% in specifications 3.1, 3.2, and 3.3. While this threshold, which is commonly used in economics, may be considered a low benchmark, please also note that our framework involves only 200 observations, and thus this statistical threshold seems to be more than adequate.

**Table 3:** F-GLS Religious ratio (2020/2018) random effect

	(3.1)	(3.2)	(3.3)
	RatioRel	RatioRel	RatioRel
Cases	0.00000405*	0.00000404*	0.00000404*
	(1.72)	(1.70)	(1.70)
Deaths	−0.000125	−0.000125	−0.000125
	(−1.35)	(−1.34)	(−1.34)
DLom	−0.229	−0.208	−0.208
	(−0.74)	(−0.66)	(−0.66)
Digital divide		0.00514	0.00514
		(0.43)	(0.43)
DEas			0.335***
			(2.78)
Dummy week	Yes	Yes	Yes
Constant	0.173*	−0.0193	−0.0193
	(1.91)	(−0.04)	(−0.04)
Observations	200	200	200

*t* statistics in parentheses *p < 0.1, **p < 0.05, ***p < 0.01

Interestingly enough, the number of deaths seems to have no effect on religiousness. The number of cases drives the dynamic of increased religiousness: not the deaths. This may be due to fear and the increase in uncertainty caused by the pandemic and its spread, rather than the actual count of deaths. These findings together suggest that an increase in COVID-19 cases has indeed caused the population to become more religious than it used to be.

Finally, the operationalization of technological backwardness, *Digital Divide*, is statistically not significant, suggesting that it does not affect this relationship. Possibly the diffusion of the Internet in Italian regions is widespread enough to support the attendance of Mass via online streaming. On the other hand, the dummy discriminating for the weeks before and of Easter, *DEas*, is positive and statistically significant at 1%, confirming the evidence that in these weeks the attendance of Mass is higher. Nevertheless, the impact of COVID-19 cases on our dependent variable *RatioRel* remains positive and statistically significant, suggesting a role played by the spread of the pandemic in this relationship.

As a robustness check, knowing that the results of a Hausman test are not definitive in deciding the best specification, we also estimate the model using Fixed Effects. The results are presented in Table 4. Please note that of course in this specification it was not possible to include either the *Digital Divide* or the *DLom* variables, since both are *region*-invariant. In this case too, the coefficient of the *Cases* variable is positive and statistically significant, suggesting that an increase in the number of COVID-19 cases makes the population of the region more religious in 2020 compared to how it was in 2018. The same reasoning already expressed also applies to the *DEas* variable, for both the week of Easter and the previous one.

**Table 4:** F-GLS religious ratio (2020/2018) fixed effect

	(4.1)	(4.2)
	RatioRel	RatioRel
Cases	0.00000581*	0.00000581*
	(1.78)	(1.78)
Deaths	−0.000188	−0.000188
	(−1.56)	(−1.56)
DEas		0.313**
		(2.50)
Dummy week	Yes	Yes
Constant	0.158*	0.158*
	(1.92)	(1.92)
Observations	200	

*t* statistics in parentheses, *p < 0.1, **p < 0.05, ***p < 0.01

## Limitations

It is important to recognize that the present results have some limitations that should be highlighted for the reader. First, the analysis we presented is based on a quantitative methodological framework. While this of course has benefits in terms of generalization of the results, it is at the same time important to highlight that, like any research of this kind, important qualitative differences may be lost in the operationalization of complex concepts, which religiousness undoubtedly is. For this reason, it is suggested that caution be taken when extending our results to different cases.

Moreover, while the double operationalization of religiousness allows us to compare two different periods and carry out our analysis, at the same time it is important to recognize that the results are also due to this imputation, which may have its limits and may not be very precise or grasp exactly the same concept. While we believe that the regional averages used in our approach allow us to work with an interesting sample and with adequate statistical power to avoid the introduction of specific biases due to measurement error, it is not possible to remove this possibility entirely, and the reader must be duly warned.

Finally, the present research studies the Italian case, with reference to the Catholic faith. While there are no specific reasons to believe that our case study should be very different from others, at the same time extending our findings to other countries, cultures, and religions naturally has its risks.

## Conclusions

Since 2020 the world has faced a severe pandemic, which has changed and continues to change many aspects of everyday life for a large part of the global population. In this context, it is legitimate to suppose that many habits and behaviours have changed. Among these, we consider it to be of special interest how COVID-19 has affected people’s approach towards the divine, also given its importance on several economic outcomes and its entangled relationship with many other variables of economic interest.

We consider the closure of churches imposed in Italy for 10 weeks during the lockdown, and the consequent blessing by the Pope of the celebration of Mass via online streaming, to be a perfect opportunity for the exploitation of the data recently offered by Google Trends, in order to obtain a proxy of regional religious attendance during this time. By comparing these data with the most recent pre-pandemic data on regional religious attendance, and controlling for possible confounders, we manage to measure the impact of COVID-19 on religiousness, and especially on its extrinsic component.

Our analysis suggests that there was indeed an increase in religiousness due to the COVID-19 crisis, confirming some findings in the literature about religiousness and existential insecurity, and religious attitudes during natural disasters (Bentzen, 2019). Indeed, our analysis shows how Italian regions that were more affected by the pandemic crisis have seen a greater increase in their levels of religiousness (compared to levels registered before the pandemic) than regions that were less affected by the pandemic.

The increase in religiousness is driven by the number of cases, rather than by the number of deaths. It seems reasonable to speculate that what spurs religiousness is the fear of being infected, rather than the fear of dying, although of course much more precise and specific data and a different analysis would be required to test this hypothesis.

Our findings also bring into question results previously found in the literature suggesting that the COVID-19 pandemic impacts private prayer and intrinsic religion rather than churchgoing and extrinsic religion (Bentzen, 2020). As a matter of fact, our results suggest that even with churches closed, Italian attendance of Mass increased, and the relationship we have found implies this was due to the coronavirus. In short, we consider these results to be of interest, given their importance in confirming previous results on religious behaviour on the one hand, and their potential impact on religion-related economic outcomes on the other.

Further research may try to build on our findings by applying our framework to a different case, or testing whether, as was suggested for previous disasters (Bentzen, 2019), this increase holds even after the pandemic crisis passes.
